# Supplementary material for: Hidden Truchet Architecture in Zinc p‑Hydroxybenzoate
Source: J Am Chem Soc. 2026 Oct 16;148(42):46089–98. doi: 10.1021/jacs.6c15350 (PMC13721173; doi:10.1021/jacs.6c15350)
Supplement: Supplementary file 1 [file ja6c15350_si_001.pdf]

SUPPORTING INFORMATION

Hidden Truchet Architecture in Zinc p-Hydroxybenzoate

Hunter J. Windsor, Guy Greenbaum, Tristan N. Dolling, Yevheniia Kholina, and Andrew L. Goodwin*

Department of Chemistry, University of Oxford, Inorganic Chemistry Laboratory, Oxford OX1 3QR, U.K.

E-mail: andrew.goodwin@chem.ox.ac.uk

Contents

S1 Synthesis **S3**

Previously reported method for Li(inox) S3

Previously reported method for Zn(hba) S3

Zn(hba) S3

Zn(hca) S4

Crystallisation of H_2hca S4

S2 Single Crystal X-Ray Diffraction **S5**

Refinement S5

S3 Voronoi Decomposition **S12**

S4 Diffuse Scattering and 3D-ΔPDF Analysis **S13**

S5 Powder X-Ray Diffraction **S17**

S6 Monte Carlo Simulations **S18**

References **S20**

S1 Synthesis

All reagents were purchased commercially and used as received without further purification. Li(inox) was synthesised according to the method reported by Abrahams and Robson.[1] Zn(hba) was synthesised by a modified procedure. For reference, the previously reported synthetic method for Zn(hba) is also given below. We note that the two methods yielded single crystals suitable for X-ray diffraction with equivalent unit cells in the revised $P4_2/mmc$ setting ($a \sim 9$ Å, $c \sim 6$ Å).

Previously reported method for Li(inox)

Isonicotinic acid *N*-oxide (0.220 g, 1.58 mmol) and $LiOH{\cdot}H_2O$ (0.070 g, 1.67 mmol) were dissolved in boiling MeOH (20 mL). DMF (20 mL) was added to the hot reaction mixture. Continued heating at a temperature of 70 °C allowed the selective evaporation of the MeOH from the reaction mixture. The removal of nearly all of the MeOH from the mixture allowed needle-like crystals to separate. The crystals were dried over air before collection.

Previously reported method for Zn(hba)

n-Amyl alcohol (20 mL) was added to a methanolic solution (15 mL) containing 4-hydroxybenzoic acid (0.0622 g, 0.451 mmol) and $Zn(OAc)_2{\cdot}2\,H_2O$ (0.100 g, 0.459 mmol). The reaction mixture was heated in an open flask at 110 °C. With continued heating, the more volatile methanol was selectively lost from the reaction mixture. The removal of nearly all of the MeOH allowed the separation of colourless needle-like crystals. The crystals were collected and subsequently dried in an oven at 250 °C for 5 h.

Zn(hba)

Zinc nitrate hexahydrate (16.7 mg, 0.06 mmol) and 4-hydroxybenzoic acid (9.1 mg, 0.07 mmol) were added to a glass scintillation vial. *N*,*N*-Dimethylformamide (4 mL) was added to the

vial with dissolution of the reagents assisted by sonication. The vial was capped and heated at 115 °C for 18–24 h to yield orange block- or plate-like crystals.

Zn(hca)

Zinc nitrate hexahydrate (14.5 mg, 0.05 mmol) and *trans*-4-hydroxycyclohexanecarboxylic acid (8.1 mg, 0.06 mmol) were added to a glass scintillation vial. *N*,*N*-Dimethylformamide (3.5 mL) and ethanol (0.5 mL) were added to the vial with dissolution of the reagents assisted by sonication. The vial was capped and heated at 125 °C for 18–24 h to yield colourless needle-like crystals.

Crystallisation of H_2hca

Colourless plate crystals suitable for X-ray diffraction were grown by slow evaporation from a concentrated solution of H_2hca in ethanol.

S2 Single Crystal X-Ray Diffraction

Refinement

Single crystal X-ray diffraction data were acquired from a Rigaku XtaLAB Synergy-S Dualflex diffractometer, equipped with a PhotonJet-Cu microfocus source outputting confocal mirror-monochromated Cu-Kα radiation ($\lambda = 1.54184$ Å), and a DECTRIS EIGER 1M detector. Data were collected about ω scans in 0.5° increments and were integrated and reduced with absorption corrections being applied through CrysAlisPro (Gaussian grid face-indexed numerical integration with beam profile, or multi-scan).[2] Structure solutions were obtained by intrinsic phasing using SHELXT[3] and were refined by full-matrix least squares on all unique F^2 using SHELXL[4] as implemented within the OLEX2-1.5 GUI.[5] For Zn(hba), a solvent mask was applied through OLEX2-1.5 to account for the contribution of disordered solvent molecules to the structure factors. Molecular and crystal structure images were created using CrystalMaker 11 (Version 11.6.2).[6]

CCDC Deposition codes are as follows: Zn(hba) 2570250; H_2hca 2570251; Zn(hca) 2570252.

Table S1: Crystal data and structure refinement for Zn(hba).

CCDC number	2570250
Empirical formula	$C_{8.88}H_{8.38}N_{0.62}O_{3.62}Zn$
Formula weight	247.07
Temperature (K)	295(2)
Crystal system	Tetragonal
Space group	$P4_2/mmc$
a (Å)	9.1051(3)
c (Å)	6.3324(3)
V (Å^3)	524.97(4)
Z (Z')	2 (0.125)
ρ_{calc} (g cm^{-3})	1.563
μ (mm^{-1})	3.163
Absorption correction	Gaussian
$F(000)$	250
Crystal size (mm^3)	0.13 × 0.05 × 0.03
Radiation	Cu-Kα (λ = 1.54184 Å)
2θ range for data collection (°)	9.714 to 135.728
Index ranges	$-7 \leq h \leq 10, -10 \leq k \leq 10, -7 \leq l \leq 7$
Reflections collected	2602
Independent reflections	302 [$R_{\mathrm{int}} = 0.0162$, $R_{\mathrm{sigma}} = 0.0092$]
Data/restraints/parameters	302/42/43
Goodness-of-fit on F^2	1.082
Final R indexes [$I \geq 2\sigma(I)$]	$R_1 = 0.0589$, w$R_2 = 0.2009$
Final R indexes [all data]	$R_1 = 0.0655$, w$R_2 = 0.2122$
Largest diff. peak/hole (e Å^{-3})	0.21/−0.21

Table S2: Crystal data and structure refinement for H_2hca.

CCDC number	2570251
Empirical formula	$C_7H_{12}O_3$
Formula weight	144.17
Temperature (K)	296(2)
Crystal system	Monoclinic
Space group	$P2_1/c$
a (Å)	5.68290(10)
b (Å)	10.7950(2)
c (Å)	12.2445(2)
β (°)	90.563(2)
V (Å^3)	751.13(2)
Z (Z')	4 (1)
ρ_{calc} (g cm^{-3})	1.275
μ (mm^{-1})	0.825
Absorption correction	Multi-scan
$F(000)$	312
Crystal size (mm^3)	0.52 × 0.12 × 0.04
Radiation	Cu-Kα (λ = 1.54184 Å)
2θ range for data collection (°)	10.926 to 151.6
Index ranges	$-5 \leq h \leq 7, -13 \leq k \leq 12, -15 \leq l \leq 15$
Reflections collected	7187
Independent reflections	1503 [$R_{\text{int}} = 0.0206$, $R_{\text{sigma}} = 0.0188$]
Data/restraints/parameters	1503/0/93
Goodness-of-fit on F^2	1.054
Final R indexes [$I \geq 2\sigma(I)$]	$R_1 = 0.0394$, w$R_2 = 0.1117$
Final R indexes [all data]	$R_1 = 0.0456$, w$R_2 = 0.1182$
Largest diff. peak/hole (e Å^{-3})	0.24/−0.15

Table S3: Crystal data and structure refinement for Zn(hca).

CCDC number	2570252
Empirical formula	$C_{8.5}H_{13.5}N_{0.5}O_{3.5}Zn$
Formula weight	244.07
Temperature (K)	296(2)
Crystal system	Tetragonal
Space group	$I4_1cd$
a (Å)	18.4240(2)
c (Å)	12.4115(2)
V (Å^3)	4213.01(11)
Z (Z')	16 (1)
ρ_{calc} (g cm^{-3})	1.539
μ (mm^{-1})	3.119
Absorption correction	Gaussian
$F(000)$	2016
Crystal size (mm^3)	0.09 × 0.05 × 0.04
Radiation	Cu-Kα (λ = 1.54184 Å)
2θ range for data collection (°)	9.6 to 151.31
Index ranges	$-21 \leq h \leq 23, -17 \leq k \leq 21, -8 \leq l \leq 15$
Reflections collected	5884
Independent reflections	1584 [$R_{\mathrm{int}} = 0.0228$, $R_{\mathrm{sigma}} = 0.0214$]
Data/restraints/parameters	1548/35/147
Goodness-of-fit on F^2	1.034
Final R indexes [$I \geq 2\sigma(I)$]	$R_1 = 0.0327$, w$R_2 = 0.0865$
Final R indexes [all data]	$R_1 = 0.0339$, w$R_2 = 0.0883$
Largest diff. peak/hole (e Å^{-3})	0.59/−0.33
Flack parameter	0.05(8)

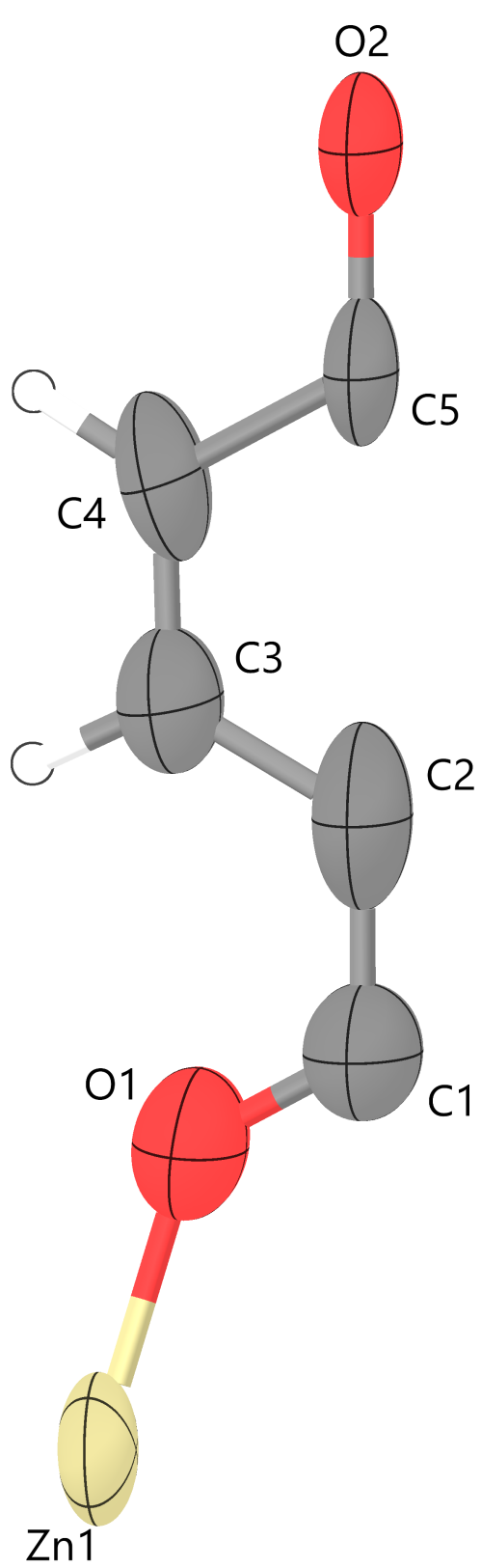

Figure S1: Asymmetric unit of Zn(hba) from our redetermined crystal structure. Displacement ellipsoids are drawn at the 50% probability level. H atoms are shown as small spheres of arbitrary radius.

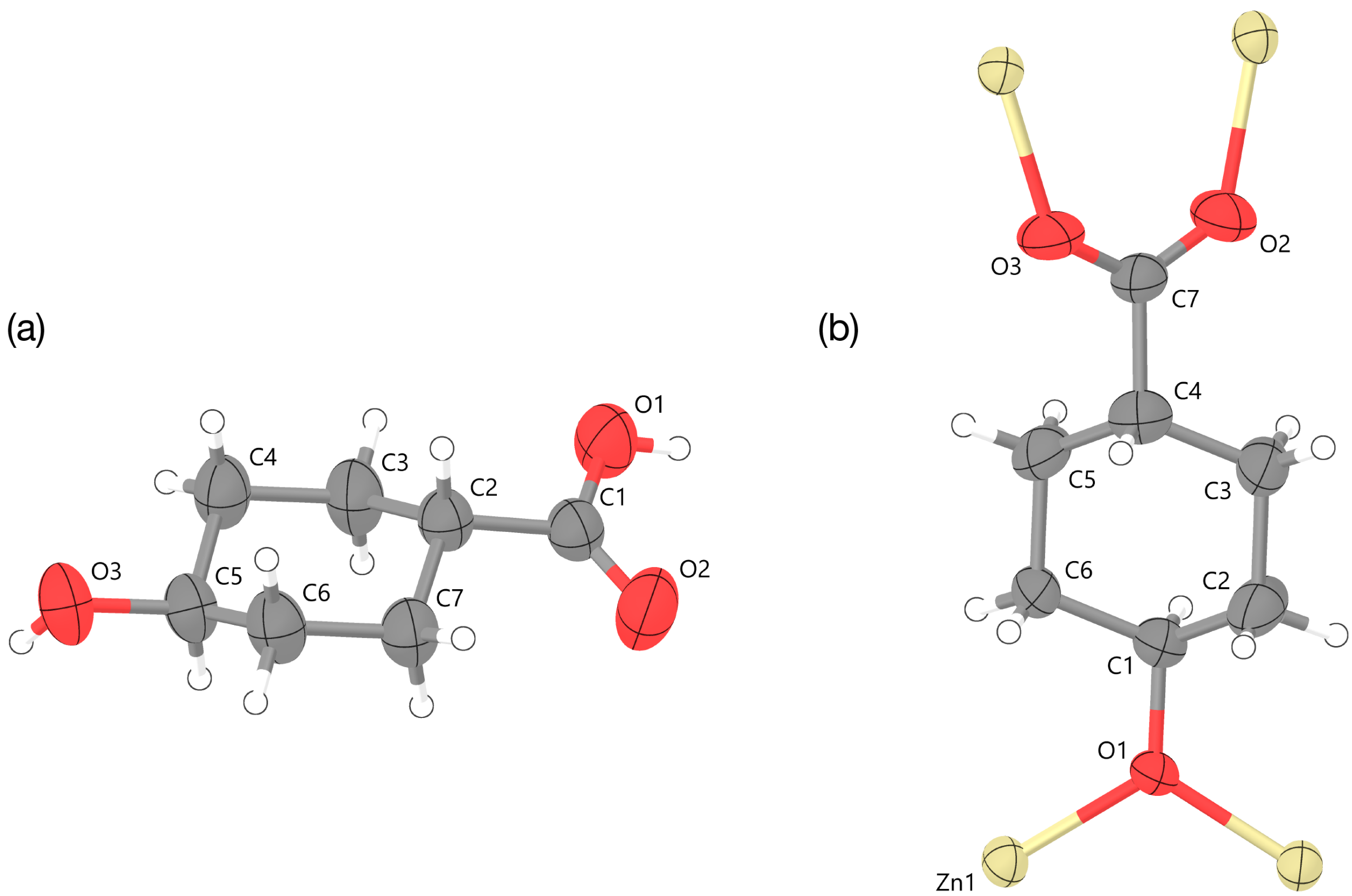

Figure S2: (a) Molecular structure and asymmetric unit of H_2hca. (b) Fragment of the Zn(hca) crystal structure with labelled atoms corresponding to those in the asymmetric unit and autoincluded DMF solvent molecule omitted for clarity. Displacement ellipsoids are drawn at the 50% probability level in both figures. H atoms are shown as small spheres of arbitrary radius.

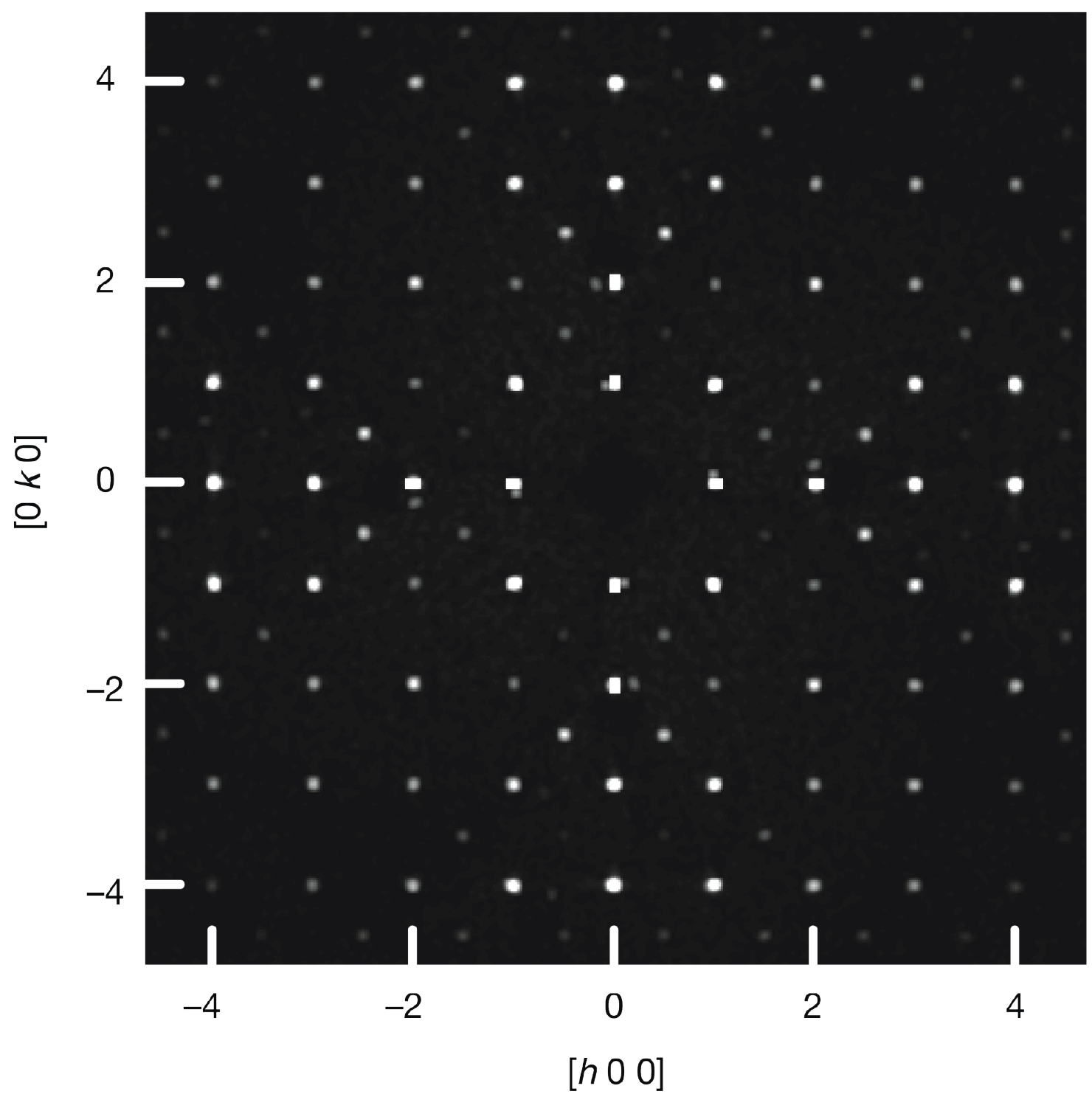

Figure S3: $hk0$ slice of reciprocal space for Zn(hca), noting the lack of structured diffuse scattering. Additional (weaker) reflections are due to a minor twin component.

S3 Voronoi Decomposition

A $3 \times 3 \times 3$ supercell configuration of the average node and linker positions in each of Li(inox) and Zn(hba) were generated using CrystalMaker.[6] This supercell size ensured that the central cell was entirely embedded within the configuration. The corresponding node and linker positions were converted from relative to cartesian coordinates (in Å) using the cell vectors. The resulting coordinates were then used as input for the *voronoin*[7] function in MATLAB. This routine generates the vertices of Voronoi cells from a given set of input coordinates, but does not support periodic boundary conditions (hence our use of an embedded cell). The resulting Voronoi vertex coordinates were back transformed from cartesian to cell coordinates, and those coordinates contained within the central cell of the supercell identified. These vertex coordinates were reduced to a set of symmetry-distinct coordinates by applying the relevant crystallographic symmetry within CrystalMaker and discarding duplicates.

S4 Diffuse Scattering and 3D-ΔPDF Analysis

Whereas Bragg scattering encodes the long-range periodic average structure, structured diffuse scattering arises from correlated local deviations from that average. The diffuse component of the scattered intensity is given by

$$I_{\text{Diffuse}}(\boldsymbol{Q}) = I_{\text{Total}}(\boldsymbol{Q}) - I_{\text{Bragg}}(\boldsymbol{Q}), \tag{1}$$

where $\mathbf{Q}$ is the three-dimensional scattering vector. The Fourier transform of $I_{\text{Diffuse}}(\boldsymbol{Q})$ is the three-dimensional difference pair distribution function (3D-ΔPDF),[8] which corresponds to the difference between the pair distribution function of the real, locally-correlated structure and the Patterson function:

$$\text{3D-}\Delta\text{PDF} = \text{FT}[I_{\text{Diffuse}}] = \langle \rho(\boldsymbol{r}) \star \rho(\boldsymbol{r}) \rangle - \langle \rho(\boldsymbol{r}) \rangle \star \langle \rho(\boldsymbol{r}) \rangle. \tag{2}$$

The 3D-ΔPDF captures those pair correlations responsible for the diffuse scattering. Positive (negative) signals at a position uvw indicate that interatomic vectors (u, v, w) occur more (less) frequently in the real structure than would be expected from the average structure in the absence of correlations.

Single-crystal diffuse scattering datasets were measured using the same Rigaku XtaLAB Synergy-S diffractometer and Cu-Kα wavelength described in Section S1.2, with each dataset collected as a single 360° φ-scan in 0.2° increments. Three-dimensional reciprocal-space maps were reconstructed from these data using a custom-written reconstruction script relying on the orientation matrix determined in CrysAlisPro.[2] The reconstructed volume spanned ± 6 in h, k, and l on a grid of $601 \times 601 \times 601$ voxels. Subsequent processing was carried out using the program *meerkat-average*;[9] the spherically symmetric background was subtracted, and the dataset was averaged in the $4/mmm$ Laue class to improve counting statistics, after which the Bragg peaks were removed using a punch-and-fill procedure in which the intensity

within a sphere of 6 voxels centred on each reciprocal-lattice point was excised and filled with values interpolated from the surrounding intensity distribution. The resulting diffuse-only dataset was Fourier transformed to yield the 3D-ΔPDF maps shown in Fig. S4.

The 3D-ΔPDF maps provide a direct real-space visualisation of the correlations inferred from the diffuse scattering in the main text. In the $uv0$ section [Fig. S4(a)], the signal is concentrated along the u and v axes, reflecting the strong one-dimensional correlations along the $\langle 100 \rangle$ ligand-row directions; the comparatively rapid decay of the signal away from these axes reflects the weakness of correlations between neighbouring rows. In the $u0w$ and uuw sections [Fig. S4(a,b)], the sign of the signal alternates with successive integer values of w, reflecting the strong tendency of ligand-row polarisations to invert from one (a, b) layer to the next along $\mathbf{c}$.

In Figure 3(b) of the main text we show the rocking curve for the $[50l]$ axis of the experimental X-ray diffraction pattern of Zn(hba), which makes obvious that the characters of the integral and half-integral l peaks are different from one another. We found that the integral (Bragg) peaks are well described by a Gaussian function centred at the Bragg position, but that the peak-shape at the half-integral (diffuse) position was better described by a Lorentzian function. The existence of Lorentzian curvature is characteristic of short-range correlations, since the Fourier transform corresponds to an exponential decay function with a characteristic decay length that is inversely proportional to the Lorentzian peak-width. In principle, one expects that a Voigt (or pseudo-Voigt) profile would provide an even better description of these diffuse features, but we have relatively few data points in some cases (since the diffuse features are so weak), and so have restricted our analysis to a Lorentzian function.

We show in Figure S5 the fits to rocking curves obtained for two parallel cuts $[40l]$ and $[50l]$ in the diffraction pattern. The corresponding full widths at half maximum obtained from our fits to data for these two cuts are given in Table S4.

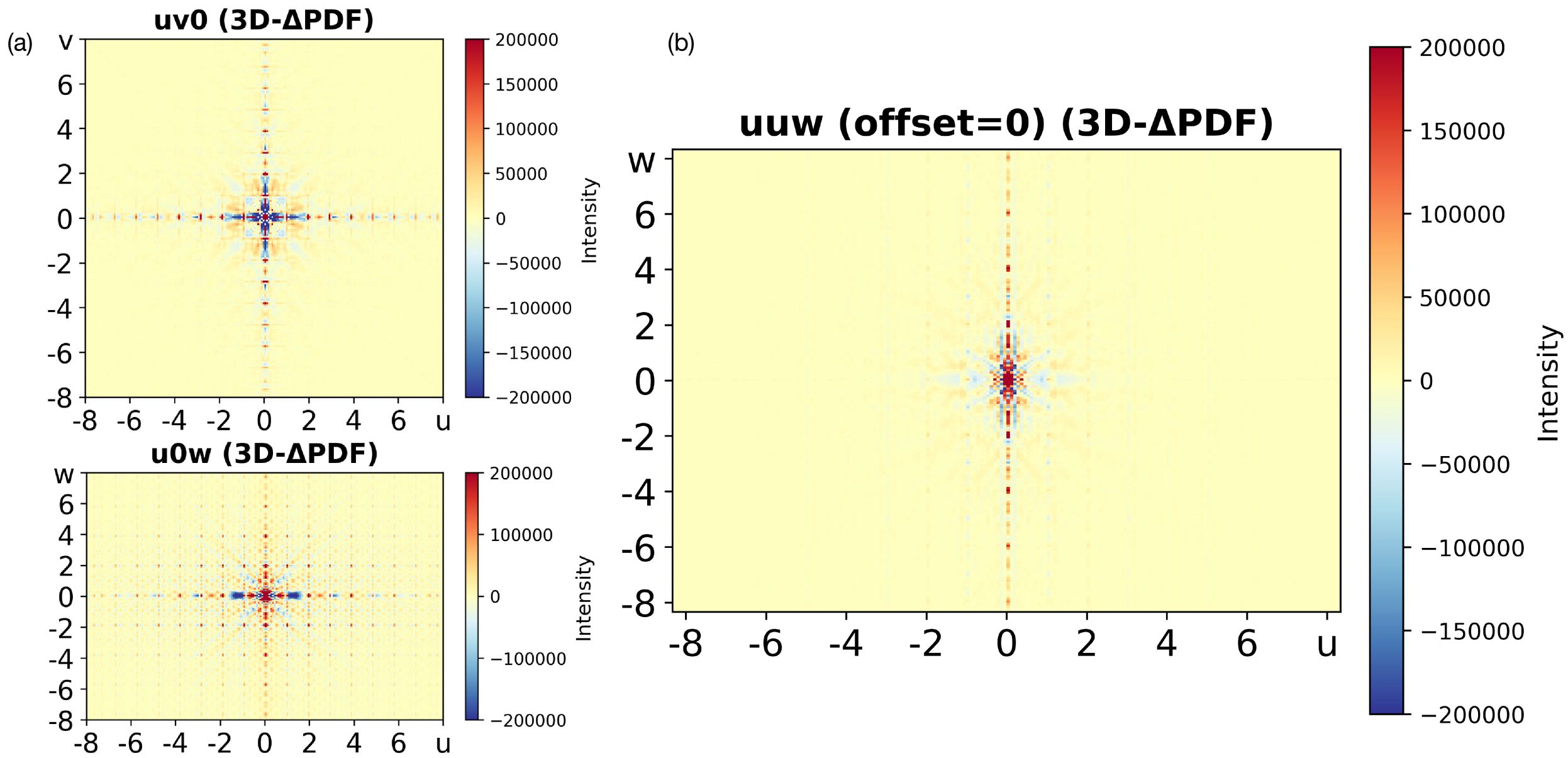

Figure S4: 3D-ΔPDF maps of Zn(hba), calculated by first removing the Bragg peaks from the reconstructed diffuse scattering dataset via a punch-and-fill procedure and then Fourier transforming the remaining diffuse scattering. The maps comprise (a) the $uv0$ (top) and $u0w$ (bottom) cross-sections and (b) the uuw cross-section, taken through the origin. Positive (red) signals correspond to interatomic vectors that occur more frequently in the real structure than in the average structure; negative (blue) signals correspond to vectors that occur less frequently. The concentration of signal along the u and v axes in the $uv0$ section reflects the one-dimensional correlations along the $\langle 100 \rangle$ ligand-row directions, with only weak correlations between neighbouring rows. In the $u0w$ and uuw sections, the alternation in sign of the signal with successive values of w reflects the strong tendency of neighbouring (a, b) layers to adopt inverted ligand-row polarisations.

Table S4: Extracted full-width-half-maxima for Bragg and diffuse peaks as determined by fits to the experimental $[40l]$ and $[50l]$ rocking curves ($-1 \leq l \leq 1$). Values are given in reciprocal lattice units.

Rocking curve	Bragg	Diffuse
$[40l]$	0.0599(15)	0.087(7)
$[50l]$	0.0308(4)	0.0599(15)

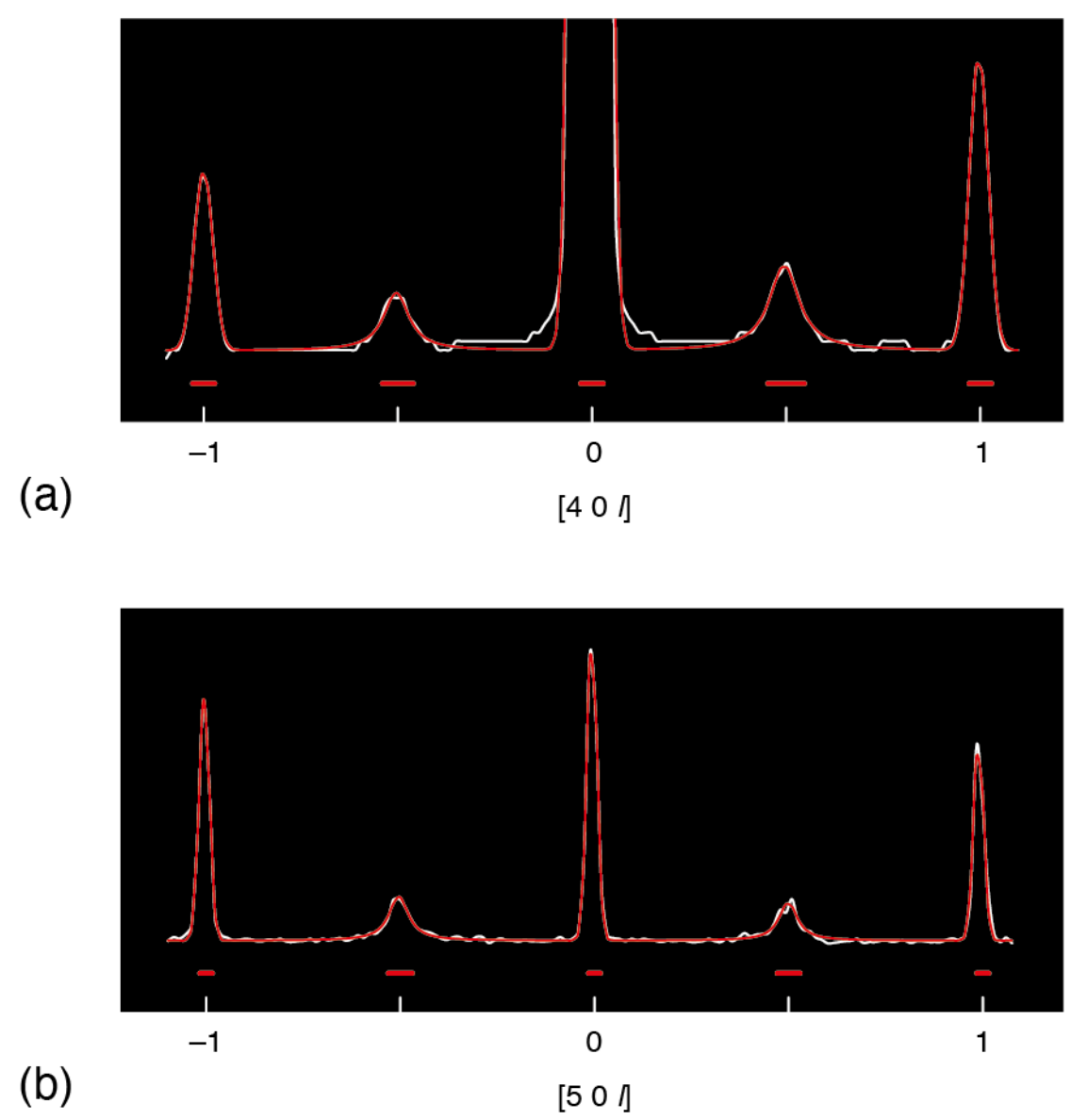

Figure S5: Extracted rocking curves for (a)[$40l$] and (b) [$50l$] cuts of the experimental X-ray diffraction pattern of Zn(hba). That shown in (b) is precisely that given in Fig. 3(b) of the main text. Data are shown as white lines. These data are fitted using a function comprising five independent single-peak functions, where those at integral l positions are Gaussians and those at half-integral l are Lorentzians. The fits are shown as red lines, and the corresponding full-width half maxima are indicated below each peak as horizontal lines. Note that in (a) the diffraction intensities are very low; hence the (400) peak itself is truncated vertically.

S5 Powder X-Ray Diffraction

Room temperature powder X-ray diffraction patterns were acquired from a Bruker D8 Advance Eco diffractometer operating in Bragg–Brentano geometry, outputting Cu-Kα ($\lambda = 1.5418$ Å) radiation, and equipped with a LYNXEYE XE-T silicon detector. Single crystalline samples of Zn(hba) or Zn(hca) were isolated from their respective crystallisation mother liquors and washed with *N*,*N*-Dimethylformamide (3×5 mL) to remove any residual ligand or metal salt. The samples were then gently ground to produce polycrystalline powders at which point air-drying at 80 °C was carried out until the point where the majority of surface-bound solvent was removed. Data were acquired over a 2θ range of 5–50°.

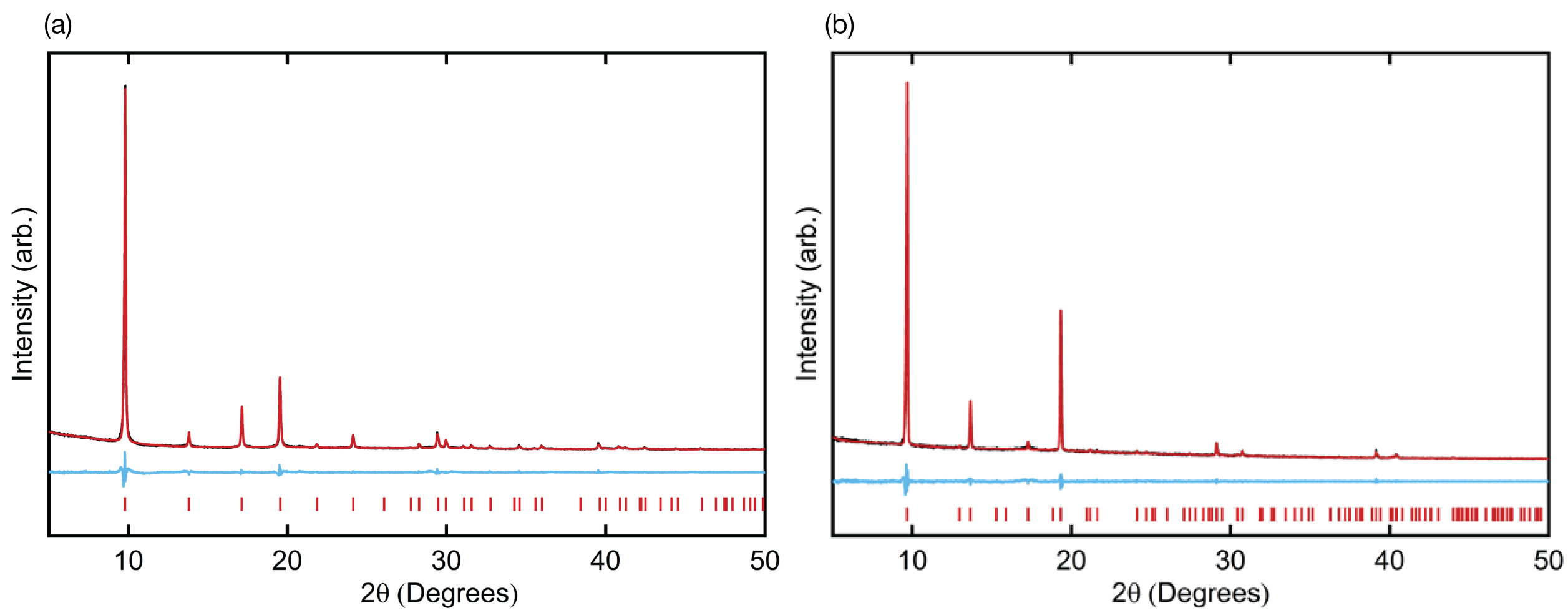

Figure S6: Pawley fits to the room temperature PXRD patterns of (a) Zn(hba) [refined unit cell parameters: $a = 9.13625(5)$ Å; $c = 6.33397(5)$ Å; $R_{\mathrm{wp}} = 7.688\%$] and (b) Zn(hca) [refined unit cell parameters: $a = 18.4429(9)$ Å; $c = 12.4424(6)$ Å; $R_{\mathrm{wp}} = 6.716\%$]. Colour code: data (black), fit (red), difference function (blue), and calculated reflection positions (dark-red tick marks).

S6 Monte Carlo Simulations

We used a two-step Monte Carlo (MC) approach to generate representative atomistic models of Zn(hba). The first step focused on generating a configuration with suitable orientations of hba linkers. The polarisation of each chain (row) of hba ligands within a single (a, b) layer was treated as an Ising variable $p_i = \pm 1$. The row polarisations interacted according to Eq. (1) of the main text, where the sum is taken over neighbouring parallel rows and $J > 0$ favours antipolar correlations. Simulations were performed for an 8×8 layer with periodic boundary conditions. Trial moves consisted of reversing the polarisation of a randomly-selected row and were accepted or rejected according to the Metropolis criterion. Each simulation was initialised from a random configuration of row polarisations, and subsequently annealed from an effective Monte Carlo temperature $T_{\mathrm{MC}} = 20J$ to $2J$. The temperature was multiplied by a factor of 0.95 after every 512 attempted moves. Twenty independent annealing runs were performed. The final two-dimensional configurations were expanded along the crystallographic $\mathbf{c}$ direction by reversing all row polarisations between successive layers. Seven layers were used to form a $8 \times 8 \times 7$ configuration. The odd number of layers introduced a single stacking fault into the otherwise antipolar stacking sequence, mimicking the $\sim 50\,\text{Å}$ correlation length inferred from the experimental diffuse scattering. The resulting set of linker orientations was then translated into an atomistic model by decorating with hba ligand coordinates, and the Zn^{2+} positions were assigned according to the orientations of the intersecting ligand rows.

In the second step we allowed for thermal displacements. The hba ligands were treated as rigid bodies, and the energy was described by harmonic Zn–O bond potentials as given in Eq. (2) of the main text. Equilibrium distances of 1.951 and 2.012 Å were used for the carboxylate and phenoxide Zn–O bonds, respectively. A common force constant of $3\,\text{eV}\,\text{Å}^{-2}$ was used for both bond types as this was found to produce physically sensible displacements. Trial moves consisted of either a translation or a rotation. For translations, a randomly selected Zn^{2+} ion or hba ligand rigid body was displaced independently along each Cartesian direction by an amount selected uniformly from the interval $[-1, 1]\,\text{Å}$. For rotations, a

randomly selected hba ligand was rotated about a randomly oriented axis passing through its centre, with a maximum rotation angle of 5°. Periodic boundary conditions were applied, and the unit-cell parameters were held fixed. Moves were accepted or rejected according to the Metropolis criterion. Each simulation consisted of 5×10^6 attempted moves. Approximately 20 independent relaxation runs were performed for each of 20 input configurations, and the resulting 347 structures were used for the diffuse-scattering calculations.

References

(1) White, K. F.; Abrahams, B. F.; Babarao, R.; Dharma, A. D.; Hudson, T. A.; Maynard-Casely, H. E.; Robson, R. A New Structural Family of Gas-Sorbing Coordination Polymers Derived from Phenolic Carboxylic Acids. *Chem. Eur. J.* **2015**, *21*, 18057–18061.

(2) Rigaku OD *CrysAlis PRO*. Rigaku Oxford Diffraction Ltd, Yarnton, Oxfordshire, England., 2025.

(3) Sheldrick, G. M. SHELXT – Integrated space-group and crystal-structure determination. *Acta Cryst.* **2015*a***, *A**71***, 3–8.

(4) Sheldrick, G. M. Crystal structure refinement with SHELXL. *Acta Cryst.* **2015*b***, *C**71***, 3–8.

(5) Dolomanov, O. V.; Bourhis, L. J.; Gildea, R. J.; Howard, J. A. K.; Puschmann, H. OLEX2: a complete structure solution, refinement and analysis program. *J. Appl. Cryst.* **2009**, *42*, 339–341.

(6) Palmer, D. *CrystalMaker*. CrystalMaker Software, Bicester, Oxfordshire, England. https://www.crystalmaker.com/, 2020.

(7) Barber, C. B.; Dobkin, D. P.; Huhdanpaa, H. T. The Quickhull Algorithm for Convex Hulls. *ACM Trans. Math. Softw.* **1996**, *22*, 469–483.

(8) Weber, T.; Simonov, A. The three-dimensional pair distribution function analysis of disordered single crystals: basic concepts. *Z. Krist.* **2012**, *227*, 238–247.

(9) Simonov, A. Meerkat: program for reciprocal space reconstruction. `https://github.com/aglie/meerkat`, 2019.
